# Tailored 3D Lattice SAPO-34/S-PEEK Composite Sorbents by Additive Manufacturing for Sorption Heat Transformation Applications

**DOI:** 10.3390/ma18153428

**Published:** 2025-07-22

**Authors:** Gabriele Marabello, Emanuela Mastronardo, Davide Palamara, Andrea Frazzica, Luigi Calabrese

**Affiliations:** 1 Department of Engineering, University of Messina, Contrada di Dio Sant’Agata, 98166 Messina, Italy; gabriele.marabello@studenti.unime.it (G.M.); emanuela.mastronardo@unime.it (E.M.); dpalamara@unime.it (D.P.); 2 CNR—ITAE “Nicola Giordano”, Via Salita S. Lucia sopra Contesse 5, 98126 Messina, Italy; andrea.frazzica@itae.cnr.it

**Keywords:** zeolite, composite sorbent, sulfonated polyether ether ketone, additive manufacturing

## Abstract

The development of high-performance adsorbent materials is crucial for any sorption-based energy conversion process. In such a context, composite sorbent materials, although promising in terms of performance and stability, are often challenging to shape into complex geometries. Additive manufacturing, also known as 3D printing, has emerged as a powerful technique for fabricating intricate structures with tailored properties. In this paper, an innovative three-dimensional structure, constituted by zeolite as filler and sulfonated polyether ether ketone as matrix, was obtained using additive manufacturing technology, which is mainly suitable for sorption-based energy conversion processes. The lattice structure was tailored in order to optimize the synthesis procedure and material stability. The complex three-dimensional lattice structure was obtained without a metal or plastic reinforcement support. The composite structure was evaluated to assess its structural integrity using morphological analysis. Furthermore, the adsorption/desorption capacity was evaluated using water-vapor adsorption isobars at 11 mbar at equilibrium in the temperature range 30–120 °C, confirming good adsorption/desorption capacity.

## 1. Introduction

The growing need to increase the exploitation of low-grade waste heat and renewable heat sources is leading to the investigation of innovative thermal energy storage and conversion technologies, which can also help the integration of multiple sources in complex applications [1].

In this context, thermochemical heat storage technology, based on a physical adsorption mechanism, represents a promising and viable technology, especially for long-term storage applications [2,3,4,5]. Adsorption is the process of capturing and storing molecules on the surface of a porous material. It is suitable for long-term and high-density energy storage, since the energy is stored as binding potential between the sorbent material and the working fluids (sometimes referred to as sorbate). Among various available adsorbent materials, zeolites and crystalline alumina-silicates with well-defined pore structures have attracted significant attention due to their superior adsorption properties, especially toward water vapor as sorbate [6,7].

**Figure 1:**
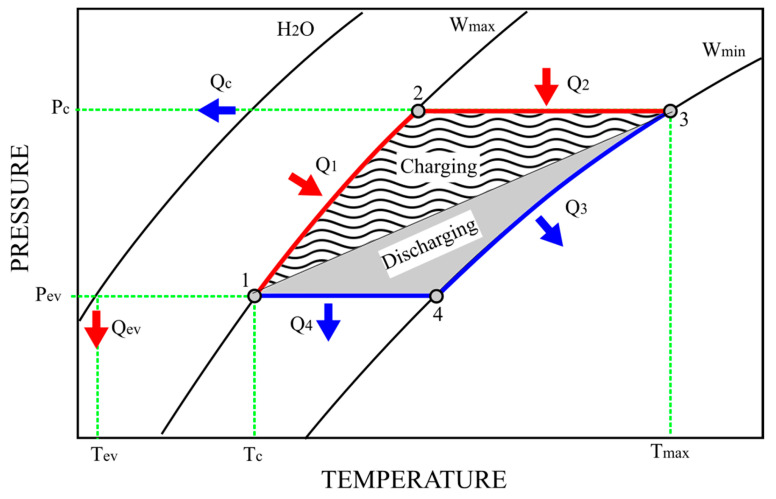
Schematic representation of a thermodynamic cycle related to an adsorption storage unit.

Figure 1 presents a schematic of the thermodynamic cycle intrinsic to an adsorption storage unit. The pedices “ev” and “c” are abbreviations for the evaporator and condenser, respectively.

The fundamental storage sorption cycle operates in two primary stages, each consisting of two unique phases. The initial stage, known as the charging phase, commences with isosteric heating (Q1), a process where heat is added to the system at a constant volume. This is immediately followed by isobaric desorption (Q2), during which the sorbent material releases the stored working fluid as vapor under constant pressure. The subsequent stage, the discharging phase, is initiated by isosteric cooling (Q3), where the system is cooled at a constant volume. This phase concludes with isobaric adsorption (Q4), where the working fluid is re-adsorbed by the sorbent at a constant pressure, completing the cycle and preparing the unit for the next charging phase.

Despite the promising features of this technology, its development at a relevant scale has been partially limited by the need for better-performing materials and suitable technologies for the adsorbent material integration and shaping into final compact and efficient components. Indeed, the utilization of zeolites in sorption thermal energy storage and conversion applications is usually hindered by the contemporary need to maximize the heat and mass transfer performance inside the sorption-based energy system [8].

To overcome these limitations, researchers have explored the fabrication of composite materials incorporating zeolites with matrices that enhance their mechanical properties. The first attempts were focused on the realization of thin structures coating the metallic surface of heat exchangers to maximize the heat transfer efficiency by avoiding excessive mass transfer limitations [9,10,11]. This approach demonstrated the ability to achieve very high specific cooling power, overcoming the state-of-the-art loose grains adsorber by more than 30%. Accordingly, many investigations were conducted aiming at the optimization of the binder and the overall composition of the coating. Specifically, silane [12], silicone [13], and sulfonated polyether ether ketone [14] were proposed and thoroughly analyzed for heat pumping, desalination, and desiccant applications. The obtained results showed promising features in terms of performance and stability of the different configurations. Nevertheless, the limited amount of adsorbent material per unit of volume due to the thin coated structure, lower than 500 µm, indicated that they were not suitable for heat storage applications. Indeed, in sorption heat storage technology, the overall energy storage capacity is the primary performance indicator and depends directly on the amount of adsorbent material embedded inside the reactor. For this reason, more recently, a novel composite material based on a matrix and embedded zeolite was proposed. It is based on a porous polymeric foam, which is able to fill the entire volume of the heat exchanger, thus maximizing the volumetric adsorption capacity of the storage reactor [15]. This approach was also successfully proposed to embed other active phases, such as salt hydrates, with higher performance in sorption heat storage applications [16].

Building on the previous experience, a novel concept is herein proposed based on additive manufacturing processes to create complex 3D structures. It exploits sulfonated polyether ether ketone (S-PEEK) as a polymer for composite manufacturing. S-PEEK, a high-performance thermoplastic polymer, has emerged as a promising matrix material due to its high strength, thermal stability, and water-vapor diffusion [17]. The combination of zeolite and S-PEEK holds the potential for developing advanced adsorbent materials for energy storage and conversion applications.

In previous works [14,18], a composite material was proposed consisting of a high content of zeolite (up to 90% by weight of SAPO-34) and a sulfonated polyether ether ketone (S-PEEK) binder. The S-PEEK matrix has high water-vapor permeability, thereby minimizing mass diffusion limits within the adsorbent composite bulk. Furthermore, this composite material has shown promising results in terms of mechanical, thermal, and ad/desorption stability [19]. Furthermore, this polymer is also notable for its good thermal stability, with thermal degradation commencing only at approximately 340 °C, specifically linked to the desulfonation reaction of its SO_3_H groups [19]. Consequently, this high degradation temperature confirms the polymer’s suitability within the typical operating temperature range encountered in most sorption technologies, making it a reliable material for such applications.

Despite the interesting results achieved, as already introduced, the coating methodology proposed has some limitations in terms of the amount of material to be loaded inside the adsorber reactor as well as the difficulty in coating complex geometries. For these reasons, the exploitation of innovative additive manufacturing methodologies can help in obtaining the most effective integration of composite sorbent structures with highly complex designs inside any kind of heat exchangers.

Additive manufacturing (AM), also known as 3D printing, provides a versatile and precise approach for creating complex structures with intricate geometries. Advances in additive manufacturing (AM) technology have paved the way for the creation of complex 3D adsorbent lattice structures, opening doors to enhanced performance in solid sorption chillers without compromising efficiency or power density. These lattice structures, meticulously engineered through AM, offer unparalleled optimization of heat and mass transfer, propelling these chillers closer to their true potential [20]. So far, in the literature, only a few examples of AM application in the field of sorption technologies for heating, cooling, and storage have been reported. In [21], the application of 3D printing techniques for different sorption processes is discussed, particularly focusing on the optimization of heat and mass transfer thanks to the triply periodic minimal surface structure configuration. More specifically, the same authors in [22] describe in detail the activity performed with a 3D-printed structure for water harvesting. The experimental results show the doubling of both energy efficiency and water productivity when the innovative 3D-printed structure is compared against a conventional configuration. This demonstrates the potential of this technology when complex structures need to be integrated into sorbent reactors. Similar studies were presented for other reactor designs, employing both novel adsorbent materials (e.g., MOFs) and standard ones (e.g., zeolite 13 X) for direct air capture and CO_2_ adsorption applications [23,24].

The ability to tailor the pore structure and composition of the lattice structure provides flexibility for optimizing the adsorbent properties for specific applications.

The integration of 3D adsorbent lattice structures into sorption-based systems, such as adsorption storage, chillers, and heat pumps, has the potential to revolutionize energy management and utilization [25,26]. These systems can provide efficient and sustainable cooling and heating solutions for buildings and industrial processes, reducing reliance on fossil fuels and minimizing environmental impact.

The ability of 3D printing to create complex geometries offers significant advantages for sorption applications compared to traditional forms like honeycomb structures, conventional coatings, or packed beds. A cuboctahedra lattice, for instance, allows for tailored internal pathways that can significantly reduce diffusion resistance for water vapor, accelerating adsorption kinetics. Unlike randomly packed beds, the distributed and interconnected porosity of a 3D lattice minimizes the less effective areas and ensures uniform vapor distribution throughout the sorbent material. Crucially, these complex designs also enable optimized thermal transfer. The increased surface area-to-volume ratio and interconnected channels within a 3D lattice facilitate more efficient heat exchange during adsorption and desorption cycles in the composite material, which is relevant for system efficiency. Honeycomb structures, while offering some advantages, typically have less customizable internal connectivity and heat transfer paths compared to the truly 3D-interpenetrating networks achievable with additive manufacturing, providing a superior balance of mass and heat transfer for enhanced overall performance.

In this context, the present paper aims to investigate the use of this material for the realization of three-dimensional lattice structures entirely made of adsorbent composite material (without metal support) with the aid of the additive manufacturing technique for sorption-based conversion technology applications. The composite sorbent material is made of SAPO-34 zeolite and sulfonated polyether ether ketone as filler and matrix, respectively. The lattice structure is tailored for optimal synthesis, material stability, and adsorption/desorption capacity. A complex three-dimensional lattice structure was synthesized without conventional metal support. Its structural integrity was evaluated through morphological characterization. Adsorption/desorption performance was evaluated using water-vapor adsorption isobars at 11 mbar and 30–120 °C, demonstrating excellent adsorption/desorption capabilities.

## 2. Materials and Methods

### 2.1. Materials

The polyether ether ketone (PEEK) polymer (glass transition 143–160 °C, melting range 338–348 °C), 0.5–1 mm granules, was supplied by HEROFLON Italy (Collebeato (BS), Italy), H_2_SO_4_ (98% purity) was acquired from Sigma-Aldrich (St. Louis, MO, USA), and N,N-dimethylformamide, DMF (HCON(CH_3_)_2_, 99.8% purity) was obtained from Honeywell. A porous, microporous SAPO-34 zeolite (AQSOA Z02) manufactured by Mitsubishi Plastics Inc. (Tokyo, Japan) was employed as an absorbent filler with particles ranging from 5 to 10 µm in diameter.

The sulfonation of PEEK was performed as described previously [18]. Briefly, 3 g of dried PEEK was dissolved in 25 mL of concentrated H_2_SO_4_ at 25 °C for 48 h, followed by dropwise addition into ice-cooled demineralized water to precipitate the sulfonated polymer. The precipitate was washed with distilled water until neutral and dried at 60 °C for 24 h. The degree of sulfonation (DS) of the polymer matrix was determined to be 44.9% using the titration method detailed in [18].

### 2.2. Three-Dimensional Printing Technology

The mold for the adsorbent lattice structure was created using a water-soluble filament made of polyvinyl alcohol (PVA). The molding process was conducted with the following parameters:

- Printing temperature: 180 °C
- Filling percentage: 10%
- Layer height: 0.12 mm
- Slicing software: Ultimaker CURA (version 5.7.2)
- Printing speed: 50 mm/s
- Bed temperature: 60 °C

The software used to generate the GCODE file was customized to minimize the machine nozzle’s movements or to prevent production defects on the mold’s surface. A Sidewinder x2 machine (Artillery 3D Technology Co., Ltd., Shenzhen, China) was employed, using a nozzle diameter of 0.4 mm.

The preparation of the composite S-PEEK–zeolite slurry was performed based on the procedure detailed in [27].

First, the S-PEEK polymer was dissolved in N,N-dimethylformamide (DMF) at a ratio of 1:10 under magnetic stirring for 5 min. Then, SAPO-34 powder was gradually added while continuing to stir until the target filler concentration of 80 wt.% was achieved. The resulting slurry was kept under agitation at room temperature for an additional 15 min to ensure the SAPO-34 filler was evenly distributed throughout. This is a crucial step for suitable deposition of the slurry within the PVA mold without any clots or heterogeneities, which is essential for the final product’s quality and performance. The composite slurry was added directly into the PVA mold cavities, progressively filling it, using a micro-syringe. Details of this procedure and subsequent drying step are reported in the Results and Discussion section.

### 2.3. Material Characterization

The adsorption capabilities of the composite sorbents were investigated employing a thermogravimetric dynamic vapor system (Surface Measurements Systems DVS Vacuum). The system comprises a precision micro-balance (0.1 μg sensitivity) and a water-vapor pressure flow-control device within the analytical chamber. First, the sample was degassed and its dry weight determined by heating it slowly to 130 °C (1 °C/min) and maintaining this temperature for about 6 h under continuous evacuation (10^−1^ Pa). Next, a valve was opened, connecting the evaporator (containing liquid water) to the sample chamber. Vapor then flowed through the system, with the absolute pressure maintained at 11 mbar by an automatically controlled butterfly valve and downstream vacuum pump. The polymer composite sorbent water-vapor adsorption capacity was tested under isobaric conditions at 11 mbar in the temperature range 30–120 °C. The water uptake was determined using the equation below:

(1) $$w\left(\frac{g}{g}\right)=\frac{m\left(p_{H2O},T_{S}\right)-m_{0}}{m_{0}}$$

where m(*p_H2O_,T_S_*) [*g*] is the sample’s equilibrium mass at a certain water-vapor pressure and temperature, and *m*_0_ [*g*] is the dry sample mass.

The three-dimensional-printed composite morphology was comprehensively assessed using an environmental scanning electron microscope (ESEM), specifically a Quanta 450 from FEI (Hillsboro, OR, USA). For these observations, an accelerating voltage of 10 kV was applied, and the chamber pressure was maintained at 50 Pa. The samples were analyzed as produced, requiring no additional surface preparation.

## 3. Results and Discussion

### 3.1. Design and 3D Printing of Adsorbent Lattice Structure

This section delves into the manufacturing process for the 3D-printed lattice sorbent structure, providing a comprehensive description of the key steps involved.

Lattice structures are lightweight, strong, and energy-absorbent three-dimensional structures made up of repeating cellular units. They are often made of interconnected beams (struts) and nodes, resembling three-dimensional lattices or grids. Lattice structures are ideal for minimizing material loss and energy consumption in manufacturing, preserving light weight and strength, with suitable mechanical properties and vibration-damping capabilities [28]. Additive manufacturing is the optimal method for creating such structures [29,30].

For this research project, a lattice geometry named cuboctahedron was identified. It is a geometric solid with square and triangular faces and is a common lattice structure. The cuboctahedron has eight triangular faces and six square faces, with 24 equal edges. It has cubic symmetry and 12 vertices that join triangular and square faces.

The vertices of the cuboctahedron were transformed into cylindrical structures, creating a basic cell that was then expanded into a lattice structure. The cuboctahedron’s unique geometry allowed for the creation of a lightweight, strong, and energy-absorbent lattice structure. In this configuration, the cuboctahedra form a three-dimensional cubic grid, with each cuboctahedron connected to its neighbors.

**Figure 2:**
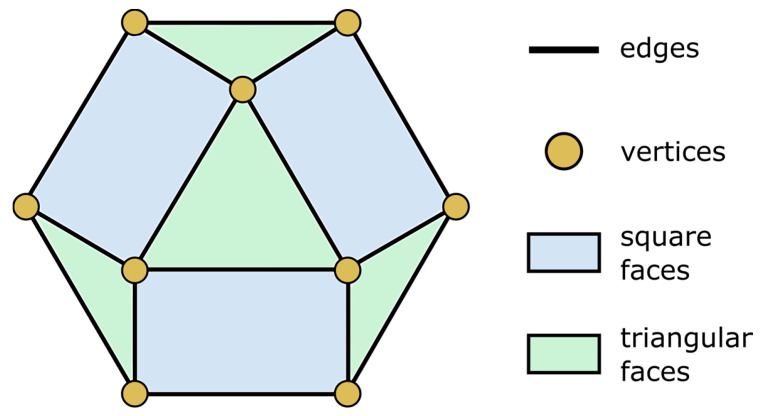
Schematic representation of a cuboctahedron with vertices, edges, and triangular and square faces.

Figure 2 shows the proposed steps for the manufacturing of the 3D-printed lattice sorbent structure. Four main steps can be identified.

**Figure 3:**
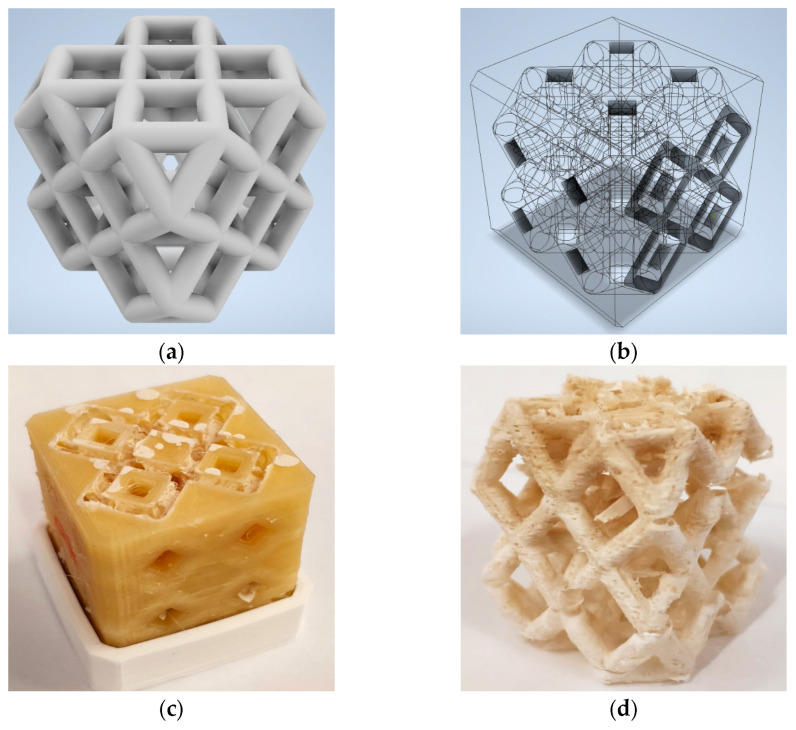
Manufacturing of the 3D-printed lattice sorbent structure: (**a**) parametric definition of the 3D cell; (**b**) negative molding of the cell; (**c**) negative molding (PVA polymer) filled with composite SAPO-34/S-PEEK slurry; (**d**) demolded lattice sorbent structure.

In Step 1, the process begins with careful CAD design, where a parametric model of the unitary lattice cell is created (Figure 3a). This spatial replica, measuring 2 × 2 × 2 in dimensions, serves as the foundation for the entire structure. Based on attempts to find the right diameter to use to pour the material into the mold, a branch diameter equal to 3 mm was defined. The design cell has dimensions of 25.686 mm × 25.686 mm × 25.686 mm. The proposed parametric model provides a standardized representation of the lattice cell, ensuring consistent dimensions and reproducibility throughout the manufacturing process. This consistency is crucial for maintaining the desired shape and properties of the sorbent structure. Furthermore, this approach is inherently flexible, allowing for easy modifications to the lattice cell design. This flexibility enables fine-tuning of the lattice structure to optimize its sorption capabilities and overall performance. Finally, a further benefit of this approach is the opportunity, in future steps, to be directly linked to numerical simulations or experimental data, enabling data-driven design optimization. This approach allows for tailoring the lattice structure to specific target molecules or applications.

In Step 2, once the lattice structure is defined, the mold is designed (Figure 3b) using Boolean subtraction between solids to obtain the negative of the structure, taking care to leave a small upper window open and leave drainage routes for the material in the lower part of the mold. A precise replica of the lattice arrangement is designed, ensuring exact adherence to the desired geometry.

In Step 3, additive manufacturing, utilizing PVA support, is employed to fabricate the negative mold, enabling the creation of a hollow lattice design. The PVA acts as a temporary support structure during composite filling, allowing for the incorporation of the solid zeolite–S-PEEK sorbent material. A counter mold is also created with the purpose of collecting the excess material that escapes from the holes on the bottom. The through-holes are also optimized to improve the dissolution process in water. The composite filling phase involves preparing a slurry comprising SAPO-34 zeolite particles and S-PEEK polymer resin. The SAPO-34 imparts exceptional sorption capabilities for targeted molecules, while the S-PEEK enhances material stability and durability. The prepared slurry is carefully dispensed into the negative mold using a micro-syringe, which ensures a uniform distribution throughout the intricate lattice structure, as depicted in Figure 3c. After depositing the slurry, we carefully dry it in an oven at a controlled temperature of 40 °C for a full day. This extended, controlled drying period is essential to ensure the complete evaporation of the DMF solvent, thus allowing the full solidification of the zeolite–S-PEEK polymer composite. Ultimately, this soft thermal treatment guarantees the material’s structural integrity and effectively prevents any cracking or deformation that could compromise its performance.

In Step 4, to liberate the solidified lattice structure from the negative mold, a dissolution procedure is employed. The mold is immersed in an ultrasonic bath at a temperature of 70 °C for a duration of 5 h. The intense ultrasonic vibrations and medium temperature effectively break down and dissolve the PVA support, allowing the lattice structure to be gently removed. The residual material left behind is the solid zeolite–S-PEEK sorbent material, retaining the precise geometry of the lattice structure (Figure 3d).

The main geometrical features of the 3D lattice composite structure produced are reported in Table 1.

In this phase of the experimental activity, we decided to create a structure characterized by a relationship between the structural and void volume to provide an extensive interconnection between solid and void channels. This was carried out in order to be able to better control both injection and demolding phases and better understand the dynamics of these application phases of the structure’s manufacturing. As reported in Table 1, the chosen 3D lattice structure is constituted by a void content equal to 66.14%. Considering that the structure created has a weight of 1.936 g and the composite material is made up of 80 wt.% zeolite, it can be deduced that the zeolite content inside the product unit is equal to 0.08 g/cm^3^.

However, these parameters can be modified during the design stage based on the geometric parameters of the 3D lattice structure (e.g., cell dimension, pipe diameter, and length) allowing tailoring of a structure with high flexibility to achieve a wide range of void content up to about 20% (80% of 3D full lattice composite material), which positively influences the maximum zeolite content per volume.

### 3.2. Morphological Characterization

**Figure 4:**
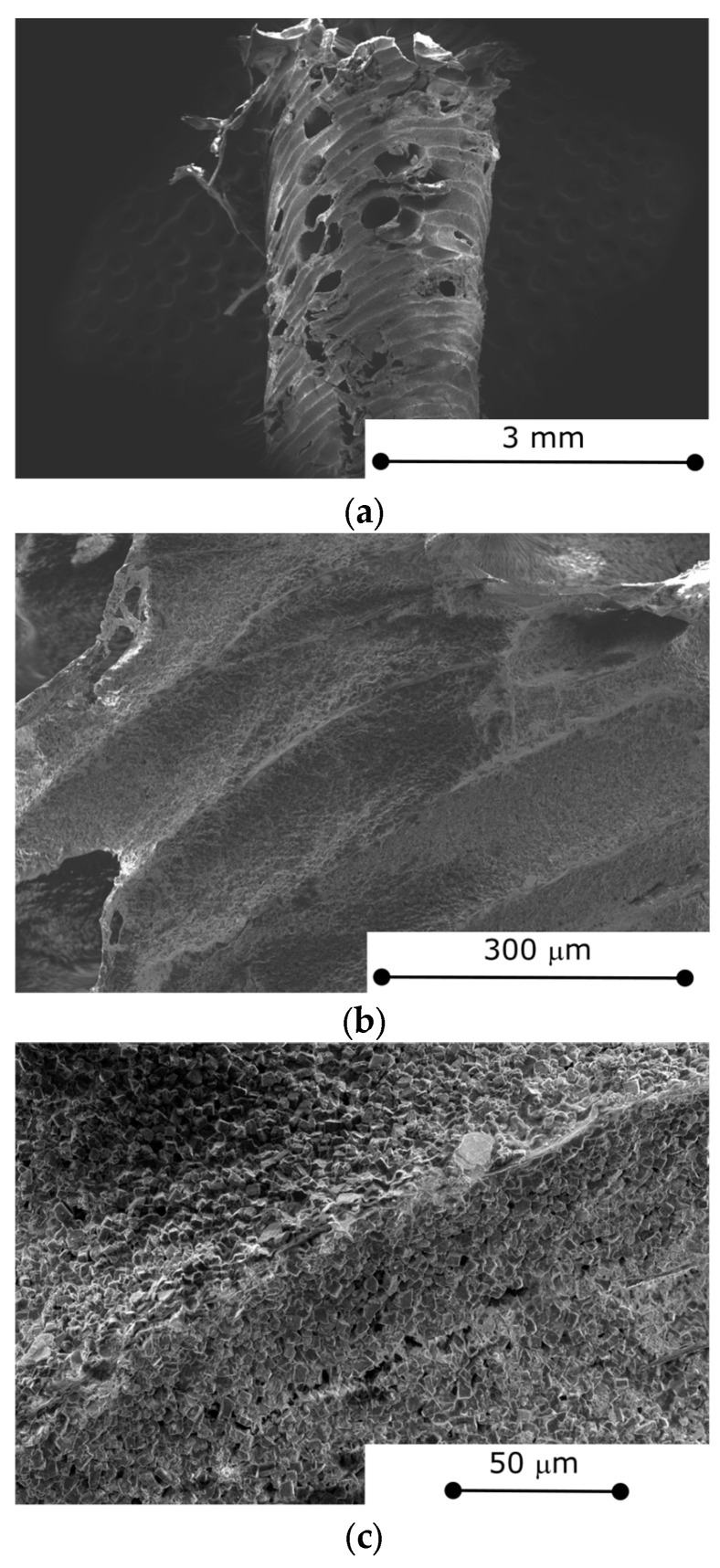
Morphological images (**a**) at low magnification, (**b**) at medium magnification, and (**c**) at high magnification of 3D-printed sorbent material.

The 3D lattice structure obtained was morphologically and structurally assessed with the purpose to robustly define the synthesized characteristics, structural homogeneity, and integrity. Figure 4a shows a detailed view of the edge of a cuboctahedron cell, which is identifiable as a thin-walled, rough tube. This particular area is notably characterized by the superficial presence of numerous holes and discontinuities. These features can be identified not as imperfections but rather as water-vapor channels between the exterior and interior of the tube. Specifically, some regions exhibit a high concentration, with approximately four holes per mm^2^, and these individual holes can reach dimensions of up to 0.4 mm. While these defects do mechanically affect the 3D structure to some extent, they do not compromise its overall stability. The inherent robustness of the 3D structure ensures it remains stable and suitable for the intended application, despite these localized imperfections.

The filler arrangement within the 3D-printed composite is uniform and well-organized, with the zeolite grains appearing to be interconnected and firmly embedded within the polymer matrix. This indicates a strong and effective interaction between the zeolite and the S-PEEK polymer. The material surface, as highlighted in Figure 4b, exhibits a somewhat rough morphology. This characteristic roughness is primarily attributed to the inherent wire layering that is an intrinsic outcome of the filament fabrication technique used in its production. This layering creates a distinctive topography of peaks and valleys that visibly line the surface, reminiscent of rivers, precisely following the molding direction of the counter-mold PVA that was employed. This contributes to the overall surface area and could influence how the material interacts with its surroundings.

Additionally, Figure 4c reveals the presence of randomly distributed micro-voids scattered throughout the composite’s bulk. Furthermore, surface and shallow cracks have also formed due to the shrinkage of the material during drying and are visible on the surface. It is important to note that despite these defects, the material stability is not compromised. Instead, these voids could play a beneficial role in identifying preferential water-vapor flow paths, thereby enhancing the adsorption/desorption dynamics.

Moreover, the high water-vapor permeability of the S-PEEK polymer, which fully surrounds the zeolite particles, hinders a possible barrier effect on the adsorption/desorption process.

Figure 4c effectively illustrates the remarkable film-forming ability of S-PEEK, which precisely replicates the intricate geometric features of the zeolite system. The S-PEEK polymer chains readily adhere to the zeolite’s surface upon contact, forming a continuous and coherent film that faithfully mimics the zeolite morphology. This indicates that the S-PEEK matrix, which acts as a binder among the zeolite fillers, has a mainly viscoelastic and flexible nature [19]. This observation suggests that the polymer matrix possesses sufficient flexibility to maintain material integrity under the thermal stresses generated during the adsorption/desorption process or in response to vibrational stresses and shocks encountered during operational life. These findings for this lattice structure offer several advantages for sorption heat transformation applications. The porous nature of the lattice provides a large surface area for adsorption, while the mechanical strength of S-PEEK and related affinity with zeolite filler ensure the structural stability of the adsorbent composite.

This intimate contact between the polymer and the zeolite ensures stable mass-transport phenomena across the composite constituents, facilitating the efficient adsorption and desorption processes that underpin thermal energy storage and conversion applications. The high degree of structural interaction between the S-PEEK film and the zeolite system enhances the performance of the S-PEEK-zeolite composite, leading to improved sorption capacity and material stability [31].

### 3.3. Adsorption/Desorption Performance

**Figure 5:**
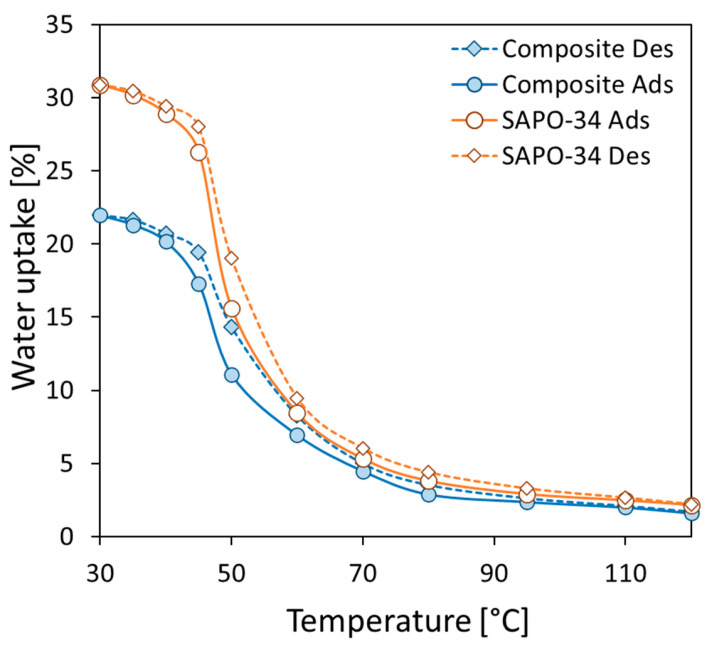
Water adsorption (circle markers—solid lines) and desorption (diamond markers—dotted lines) isobars at 11 mbar for 80 wt.% SAPO-34 zeolite filler in S-PEEK polymer material and pure SAPO-34 zeolite powder.

To evaluate the material’s potential for sorption heat transformation processes, the ability of the zeolite filler to exchange water vapor with the environment must be assessed, despite being embedded in the polymer matrix. Figure 5 illustrates the water adsorption/desorption isobars for the composite material (with 80 wt.% adsorbent filler) and pure SAPO-34 zeolite powder, evaluated at 11 mbar of water-vapor pressure. These isobars reveal the material’s ability to effectively absorb and desorb water vapor, demonstrating its potential for thermally activated sorption processes.

According to the literature [32], the SAPO-34-based composite material shows a sigmoidal shape and exhibits a sharp rise in water-vapor uptake at low temperature (T < 45 °C). Desorption isobars do not demonstrate a hysteresis phenomenon. Comparable behavior has been observed in other SAPO-34 zeolite composites [27,33]. As expected, the composite material’s water adsorption capacity is lower than that of pure SAPO-34 zeolite powder across all temperature ranges due to the reduced amount of adsorbent zeolite filler. In particular, the maximum water uptakes obtained at 30 °C for SAPO-34 powder and composite sorbents are 30.88% and 21.96%, respectively. However, the maximum water uptake observed for the composite batch is consistent with the filler content added. This suggests that the zeolite filler is effectively integrated into the polymer matrix and retains its ability to adsorb water vapor. Moreover, even if the kinetic aspects were not duly assessed at this stage, from the performed thermogravimetric equilibrium measurements discussed above, no appreciable difference in equilibrium times between the pure SAPO-34 and the 3D-printed structure was highlighted. This can indicate, only qualitatively at this stage, that the 3D printing process kept the reaction rate almost unaffected. Deeper and more dedicated investigations for the adsorption/desorption kinetics should be performed in future activities.

In this concern, a further parameter able to better assess the role of the zeolite grains inside the composite material was defined, based on the following equation:

(2) $${FZE}_{Ti}=100\cdot \left(\frac{{ZWU}_{comp}}{{WU}_{SAPO}}-1\right)$$

Filled zeolite efficiency (FZE_Ti_) is the percentage of zeolite embedded inside the adsorbent composite that contributes to the adsorption/desorption process at each temperature, Ti. Water-vapor uptake of the composite (ZWU_comp_) is the amount of water vapor absorbed by the composite normalized by the zeolite content in each sample and at each temperature, Ti, compared to the dry state. Water-vapor uptake of the pure SAPO-34 zeolite (WU_SAPO_) is the amount of water vapor absorbed by the zeolite powder at each temperature compared to the dry state. A positive FZE_Ti_ value indicates that the zeolite incorporated into the composite coating demonstrates a higher sorption capacity than the pure zeolite at that particular temperature. Conversely, a negative FZE_Ti_ value suggests that the zeolite embedded within the polymer matrix exhibits a lower sorption capacity compared to the pure zeolite powder.

**Figure 6:**
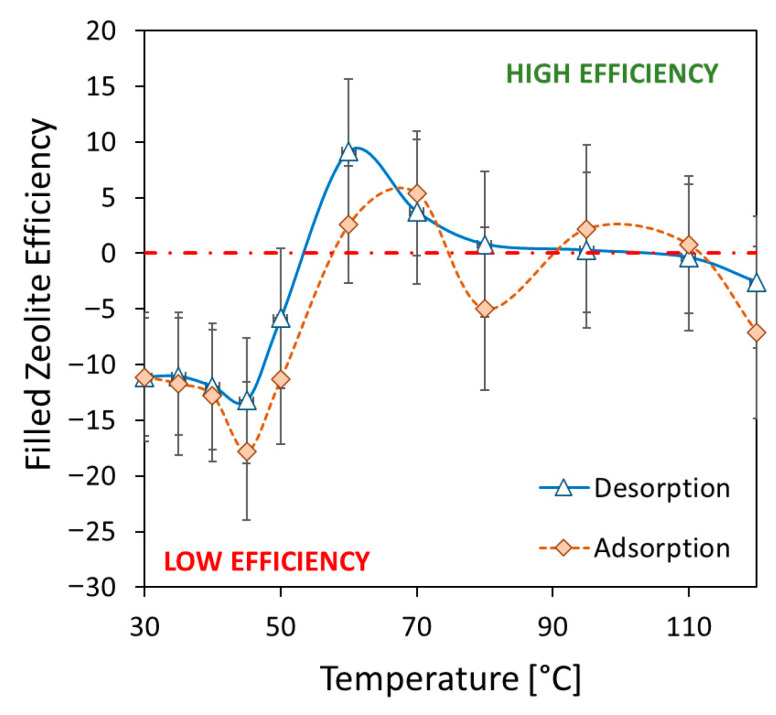
Filled zeolite efficiency at varying temperatures for adsorption and desorption of the composite material.

Figure 6 shows the filled zeolite efficiency at varying temperatures for adsorption and desorption of the composite material. The 3D-printed porous structure of the composite allows for easy vapor access throughout the material, enabling efficient water uptake even at high temperatures. The large porous channels and thin polymer films embedded with zeolites effectively facilitate the water-vapor transport, resulting in a high adsorption capacity across the entire temperature range. At low temperatures, where water uptake is maximized, the FZE index is around −10, indicating that about 90% of the SAPO-34 is actively involved in the adsorption process.

Additionally, the adsorbent lattice structure exhibits an FZE index that is near zero or slightly higher than that of pure zeolite powder. This suggests that the 3D-printed structure has an adsorption capability at higher temperatures that is almost comparable to the pure zeolite powder. The FZE data are slightly scattered because of the low sorption capacity of SAPO-34 zeolite in this range of temperatures, providing a wider and scattered range of observed filled zeolite efficiency index values. The FZE data points appear somewhat scattered because SAPO-34 zeolite exhibits a low sorption capacity within this specific temperature range. This inherent characteristic leads to a wider and more dispersed range of observed filled zeolite efficiency index (FZE) values, making the data less concentrated and more spread out.

The porous structure of the composite edges, formed by rough tubes with thin polymer films containing zeolite, allows water vapor to easily move through the entire sample, resulting in excellent adsorption capacity across a wide temperature range. However, the observed positive values for the FTE_Ti_ within this temperature range are not specifically indicative of inherently higher efficiency of the zeolite itself. Instead, this positive trend is primarily ascribed to the dispersion of the experimental data, as confirmed by error-bar distribution in this temperature range. The characteristics of the zeolite’s performance at these temperatures lead to a wider spread in the measurements, causing the FTE_Ti_ to appear positive due to this variability rather than a genuine enhancement in the material’s efficiency. This means the values reflect the scattering of data points more than a specific improvement in zeolite behavior.

At low temperatures, where water uptake is maximized, the zeolite efficiency reaches about 90%, indicating that the SAPO-34 effectively contributes to the composite’s sorption capacity. Even at high temperatures, the material’s ability to capture and hold water vapor (sorption capacity) approaches that of zeolite powder. Although this occurs, at these elevated temperatures, the forces attracting and allowing water vapor to flow through the material are significantly weakened, potentially limiting diffusion phenomena through the polymer matrix. This is because the adsorbent filler, the component responsible for capturing the vapor, has a low absorption capacity at high temperatures. However, the existence of FZE values close to zero further confirm the good vapor permeability capacity offered by the 3D-printed latex structure obtained.

Despite acting as a binder, the polymer does not interfere with the water-vapor adsorption/desorption mechanism, ensuring the material retains its efficient adsorption properties [18]. This is attributed to the S-PEEK’s remarkable water-vapor permeability [34], enabling seamless diffusion of water vapor and allowing the zeolite grains, even when fully embedded in the polymer matrix, to actively engage in adsorption and desorption processes.

These findings demonstrate the potential of this new class of materials as an alternative to traditional loose zeolite grain beds for fabricating adsorbers with superior thermodynamic adsorption performance. Future research activities will focus on investigating the hydrothermal stability and adsorption kinetics of the adsorbent composite material to comprehensively evaluate the material’s durability and assess its functionality under practical conditions.

Looking at similar composite materials reported in the literature, it can be highlighted that, in the case of foam structures where the SAPO-34 was embedded inside a permeable polymeric foam, e.g., silicone-based [19,35] [REFs], the equilibrium performance appeared comparable to the ones reported in this paper for the 3D-printed structure due to an almost linear reduction of the sorption capacity with the amount of binder used to manufacture the composite. On the other hand, as reported in [35], the foam structure, in order to guarantee a sufficient mechanical strength, requires at least 30 to 40 wt.% of binder, thus reducing the actual sorption capacity. For other composite structures, such as adsorbent coating to be deposited over heat exchangers [27,36], the results for the adsorption equilibrium are again in line with what is reported in this paper. Indeed, the adsorption isobars are almost perfectly scaled down linearly with the amount of binder employed. On the other hand, having a closer look at the efficiency of the embedded SAPO-34 in the high adsorption capacity range, it is evident that both the coating and the 3D structure have a deviation of around 10% from the theoretical maximum capacity. This effect can be attributed to the effect of the employed binder. In summary, the developed 3D-printed composite structure has good adsorption capacity in line with similar compositions previously reported in the literature.

To evaluate the impact of geometric parameters and composite material specifications on the energy storage capacities of the structure, Table 2 presents the adsorption heat values of a 3D-printed solid, whose overall geometry is defined in Table 1. Specifically, the adsorption heat, expressed in kJ, was calculated using Equation (3).

(3) $$Q_{ads}=\Delta H\cdot W\cdot \Delta w$$

To determine the heat of adsorption, the equation considered the reaction enthalpy for SAPO-34 zeolite (ΔH, 2816.6 kJ/kg [37]), the dehydrated composite material’s weight (W), and the percentage of water adsorbed (Δw) during a defined thermodynamic cycle. In particular, the adsorption heat was calculated by considering a reference working cycle, working at the same water-vapor pressure both during adsorption (i.e., evaporator pressure) and desorption (i.e., condensation pressure). This pressure was assumed to be equal to 11 mbar, corresponding to an external source/sink at 10 °C, which can be considered typical for a storage operating in winter conditions. The operating temperatures ranged from 30 °C during adsorption to 80 °C during desorption. By varying the filler content and aspect ratio while maintaining the geometry specified in Table 1, we analyzed the resulting adsorption capacities of the 3D structure. The findings, presented in Table 2, highlight the influence of material composition on performance, which is crucial for optimizing water adsorption applications.

The experimental results demonstrate a significant correlation between the filler percentage and the heat of adsorption. Specifically, an increase in filler content from 80 wt.% to 95 wt.% resulted in an approximate 60% enhancement of the heat of adsorption. Conversely, manipulating the aspect ratio from 2 to 4 yielded a substantial reduction, approximately 50%, in the heat of adsorption. This inverse relationship between aspect ratio and adsorption capacity aligns with the theoretical understanding that higher aspect ratios create a larger volume of internal voids within the 3D composite structure, which can negatively impact the material amount that effectively adsorbs target water-vapor molecules. Notably, the optimal performance was observed in a 3D structure fabricated with a composite material containing 95% filler and an aspect ratio equal to 2, which exhibited adsorption heat of 208.2 kJ. This value aligns well with results for pure zeolite, where considering a tap density of 826 kg/m^3^ and considering the whole cell volume would lead to an adsorption heat of approximately 2600 J. This consistency suggests that even within the composite, the zeolite’s inherent properties are a dominant factor in the overall adsorption characteristics, providing confidence in the observed thermal performance.

**Figure 7:**
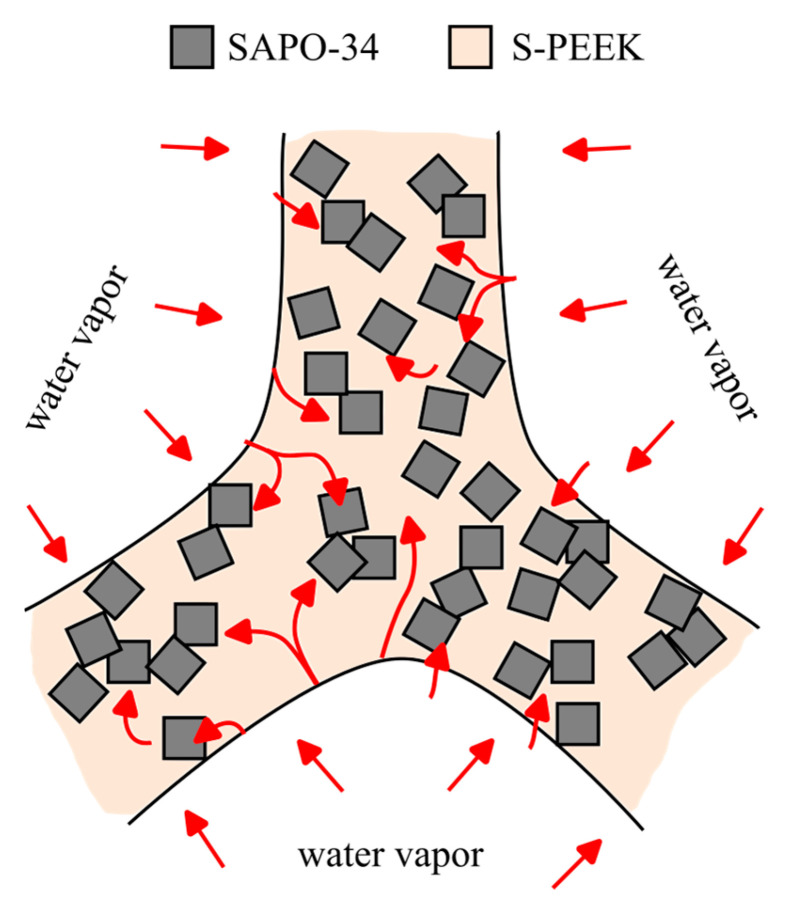
Morphological scheme of SAPO-34/S-PEEK interconnection and related water-vapor diffusion.

Based on insights from both absorption tests and morphological analysis, Figure 7 illustrates a simplified morphological scheme for the vapor diffusional flow throughout the 3D composite structure. In this model, the zeolite particles, depicted as gray filled squared markers embedded within the polymer matrix, are randomly distributed and interconnected within the composite tubes.

This arrangement implies a complex, tortuous path for vapor molecules as they move through the material, which affects overall adsorption performance. However, the zeolite filler might create preferential pathways for water vapor to diffuse within the bulk and toward the adsorbent. This is easily achieved in three-dimensional lattice structures with high zeolite content, where the tight packing suggests significant particle interconnection and potential microcavities that could facilitate diffusion. Simultaneously, since the chosen polymer matrix is permeable to vapor, diffusion can also occur along the matrix, even in areas without zeolite. This could be achieved in three-dimensional structures with lower filler content, where a higher matrix content means a more dispersed filler within the bulk. Yet, thanks to the matrix’s good vapor permeability, even zeolite particles completely enclosed by the matrix and not directly exposed to humidity can contribute to the adsorption and desorption process. This phenomenon is further aided by surface defects, voids, and the complex geometry’s tubular structure, which provides additional macro-channels to promote water vapor diffusion. These considerations are strongly corroborated by the high filled zeolite efficiency (FZE) values obtained during adsorption measurements. Notably, these values are comparable to those achieved by pure zeolite, indicating that the composite material effectively utilizes the zeolite’s adsorption capabilities despite the presence of the polymer matrix.

These findings underscore the potential efficacy of this design approach for developing tailored adsorbent structures. By precisely controlling the geometry, particularly the aspect ratio, and the material composition, specifically the filler content, it becomes possible to engineer components that are finely tuned to meet specific project requirements and operational demands.

The development of 3D adsorbent lattice structures based on SAPO-34/S-PEEK composite material using AM has opened new avenues for improving the performance of sorption-based energy systems. These lattice structures offer enhanced adsorption capacity and structural stability compared to conventional zeolite adsorbents. As research in this area progresses, 3D adsorbent lattice structures are poised to play a significant role in the development of next-generation sorption-based energy storage and conversion technologies with enhanced efficiency and sustainability.

Further research is needed to optimize the design, fabrication, and performance of 3D adsorbent lattice structures for these applications. This includes developing advanced AM techniques, exploring alternative zeolite materials, and investigating the effects of different lattice structures and operating conditions. Thanks to its flexibility in design and manufacture of sorption reactors, this methodology could be suitable both for closed and open sorption-based systems. As already reported in the literature, the first example of a sorption process that could employ such technology is represented by any open cycle working at ambient pressure, namely, open sorption thermal energy storage, water harvesting, air drying, and direct air capture. Indeed, a complex 3D structure filling a large volume would allow maximization of the sorption capacity per unit of volume, and, at the same time, working at ambient pressure, the role of mass transfer resistance across the reactor could be controlled by smart design of the internal porosity. In order to also make the technology suitable for closed working cycles operating at low pressure, more detailed simulation analysis will be needed to properly design both the structural porosity of the lattice and the thickness of the structure itself, which needs to take into account the mass transfer resistance offered toward water-vapor diffusion.

These aspects will be the objectives of further investigation and lab-scale reactor design and testing.

## 4. Conclusions

In this research, a new three-dimensional structure for sorption heat transformation was created, employing additive manufacturing technology using a composite sorbent material constituted by zeolite as a filler and sulfonated polyether ether ketone as a matrix. The lattice structure was optimized to improve the synthesis technique and material stability. The results highlighted the following:

- The interconnected three-dimensional lattice structure was created without the need for any metal or plastic reinforcement. The obtained structure was characterized by a void volume ratio equal to 66.14% and a full/composite ratio equal to 2.96.
- Morphological investigations evidenced the structural integrity of the composite structure. At the macroscopic level, the cuboctahedron cell’s edge is morphologically a rough tube with holes, representing communication channels from outside to inside the tubes. At the microscopic level, the SAPO-34 filler is well-packed and uniformly arranged in the composite, indicating suitable adhesion with the thermoplastic matrix.
- Furthermore, water vapor adsorption isobars at 11 mbar at equilibrium in the temperature range 30–120 °C were used to establish strong adsorption/desorption capability. The composite sorbent exhibited a maximum water uptake equal to 21.96% at 30 °C. The observed water uptake is consistent with the filler content. In particular, at low temperatures, the zeolite filler efficiency index is around −10, demonstrating that about 90% of the SAPO-34 is actively participating in the adsorption process.
- It was furthermore theoretically assessed that increasing the filler content (80 wt.% to 95 wt.%) enhanced adsorption heat by 60%, while raising the aspect ratio (2 to 4) decreased it by 50% due to increased internal voids reducing effective adsorption. Optimal performance was estimated with 95 wt.% filler and an aspect ratio of 2, achieving an adsorption heat equal to 208.2 kJ for the chosen geometry.

For the sake of completeness of analysis, it is also appropriate to point out that, while microscopic analysis of the material’s surface, particularly evident in the ESEM images, reveals some micro-cracks or voids, these should not compromise the structural integrity of the composite, but further clarification will be sought in future work. Furthermore, the equilibrium adsorption curves show no reduction in adsorption capacity, definitively indicating the absence of flow blockages or significant impediments to vapor diffusion within the material.

These findings demonstrate the potential of this new material class as an alternative to zeolite loose grains as adsorbers. The use of additive manufacturing to create 3D adsorbent lattice structures based on SAPO-34/S-PEEK composite material opens new paths for increasing the performance of sorption-based energy systems, potentially playing an important role in the development of next-generation devices with improved efficiency and sustainability.

This initial exploration into the structural design of 3D-printed adsorbent composites provides a crucial foundation for future advancements. Building on this, upcoming research will delve deeper into fundamental concepts like effective thermal conductivity, mass transfer limitations, and sorption dynamics. This will involve a detailed examination of how the specific geometry of the printed structure and the filler content collectively influence these critical aspects, especially as we scale toward larger, more complex 3D-printed morphologies for enhanced real-world performance. Future research will also thoroughly assess the durability and long-term functionality of these composite materials by focusing on stability and kinetics. This includes optimizing lattice design, fabrication, and performance through the exploration of advanced additive manufacturing techniques, alternative zeolite materials, and the examination of different structures and operating conditions based on the target process.

## Figures and Tables

**Table 1:** Main geometrical features of the 3D lattice composite structure.

Geometrical Feature	Value
Composite structure volume	6.59 cm^3^
3D full volume	19.46 cm^3^
Empty volume	12.87 cm^3^
Aspect ratio *	2.96
Empty volume	66.14%
Pipe diameter/length	3 mm/8.3 mm
Weight	1.936 g

* Full/composite ratio.

**Table 2:** Adsorption heat for 3D-printed structures at varying filler contents and geometries.

Filler Content wt.%	Density kg/m^3^	Δw wt.%	Aspect Ratio	Volume cm^3^	W g	Qads J
80	257.6	18.4	2	9.73	2.51	1300
			3	6.49	1.67	866
			4	4.87	1.25	650
85	288.2	20.4	2	9.73	2.80	1613
			3	6.49	1.87	1076
			4	4.87	1.40	807
90	293.5	22.4	2	9.73	2.86	1805
			3	6.49	1.90	1204
			4	4.87	1.43	903
95	310.6	24.5	2	9.73	3.02	2082
			3	6.49	2.01	1388
			4	4.87	1.51	104.1

## Data Availability

The original contributions presented in this study are included in the article. Further inquiries can be directed to the corresponding author.

## Abbreviations

The following abbreviations are used in this manuscript:

AM	Additive manufacturing
DVS	Dynamic vapor Sorption
ESEM	Environmental scanning electron microscope
PVA	Polyvinyl alcohol
RH	Relative humidity
S-PEEK	Sulfonated polyether ether ketone
T	Temperature
W	Water content
Wt.	Weight
Δw	Water-uptake variation
